# Insufficient Expression of the Autophagic Protein ATG16L1 Results in Accelerated Carcinogenesis Related to an Aberrant B Cell Response

**DOI:** 10.1002/cnr2.70438

**Published:** 2026-02-09

**Authors:** Daniela Mendiola, Betsaida Ortiz, Oscar Nieto, Marisol I. González, Danielle Vannan, Bertus Eksteen, Alejandro Martínez, Diego Mateos, Mónica G. Mendoza‐Rodríguez, Miriam Rodríguez‐Sosa, Luis I. Terrazas, José L. Reyes

**Affiliations:** ^1^ Laboratorio de Inmunología Experimental y Regulación de la Inflamación Hepato‐Intestinal, UBIMED Tlalnepantla de Baz México; ^2^ Carrera de Odontología Tlalnepantla de Baz México; ^3^ Urology Division Boston Scientific Corporation Marlborough Massachusetts USA; ^4^ Aspen Woods Clinic Calgary Alberta Canada; ^5^ Mexican Colorectal Cancer Research Consortium (MEX‐CCRC) Tlalnepantla de Baz México

**Keywords:** ATG16L1, B cells, colon cancer, IgG, oral cancer

## Abstract

**Background:**

Autophagy‐related proteins (ATGs) regulate a great variety of cellular responses beyond autophagy. In cancer, the role of ATG proteins is central, as evidenced in spontaneous cancer emerging in animals lacking ATG proteins.

**Aim:**

To determine whether ATG16L1 may be participating in tumorigenesis in colonic and oral mucosa and its likely association with adaptive immune cell deregulation (e.g., B cells).

**Methods:**

Wild‐type (WT) and ATG16L1 hypomorphic mice (ATG16L1^HM^) were induced with colitis‐associated colon cancer (CAC) by delivering azoxymethane (AOM) and dextran sodium sulfate (DSS), whereas oral cancer was generated by administering 4‐nitroquinoline‐1‐oxide (4NQO) in tap water. Tissue samples were collected and the histopathological damage was assessed. Also, secondary lymphoid organs (i.e., spleen and draining lymph nodes) were assayed for cytokine output and lymphocyte distribution by means of flow cytometry, and IgG levels were assayed in plasma samples.

**Results:**

ATG16L1^HM^ animals turned out to be more susceptible to colon and oral carcinogenesis than WT mice. In CAC, WT mice preserved colon length and presented individual colonic tumors, whereas ATG16L1^HM^ mice presented shortened colons and tumor masses. Likewise, WT mice exhibited oral leukoplakia (pre‐neoplastic lesions), whereas ATG16L1^HM^ mice showed tumors. The greater susceptibility observed in ATG16L1^HM^ animals was associated with imbalanced cytokine production (increased IL‐4 levels and lower IL‐15 output) and higher numbers of B cells in secondary lymphoid organs. This latter was also found under steady conditions, and despite having more B cells, cancer‐induced ATG16L1^HM^ mice presented lower levels of total circulating IgG.

**Conclusion:**

Suboptimal expression of the autophagic protein ATG16L1 results in accelerated carcinogenesis, and an altered B cell response may be one of the aggravating factors.

## 1 Introduction

Autophagy‐related proteins (ATG proteins) form complexes that regulate the autophagic flux. Since autophagy occurs in hematopoietic cells (leukocytes), it is now recognized that autophagy as well as individual ATG proteins are regulators of the immune response on both innate and adaptive arms [1]. Amongst ATG proteins, ATG16L1, the mammalian ortholog of yeast atg16, has emerged as a central promoter of autophagy by stabilizing the previously formed ATG5‐ATG12 complex in order to continue with the autophagosome elongation [2], but also growing evidence has revealed unexpected roles played by ATG16L1 beyond canonical autophagy, for instance, LC3‐associated phagocytosis (LAP) [3], and as a component of the complosome [4]. Further, pioneer studies unveiled that loss‐of‐function mutations in the ATG16L1 gene altered packing of lysozyme‐containing granules in Paneth cells [5] and that ATG16L1 is a break for cytokine release, evidenced by an uncontrolled output of interleukin (IL)‐1β and IL‐18 in DSS‐induced colitis in animals with conditioned deficiency of ATG16L1 in haematopoietic cells [6]. Furthermore, it has been uncovered a very close relationship between ATG16L1 and nucleotide‐oligomerization containing domain (NOD) receptors, in which ATG16L1 is critical in modulating signaling pathways involving NOD receptors and genes downstream the transcription factor nuclear factor κB (NF‐κB) such as CXCL1 [7]. Hence, ATG16L1 is recruited by NOD1 and NOD2 to be inserted in single‐membrane phagosomes participating in bacterial clearance [8]. These evidences show that ATG16L1 is required for an optimal response against intestinal commensals and partly explain why mutations in ATG16L1 are risk factors for developing inflammatory bowel diseases (IBD). Accordingly, it is now recognized that persistent inflammation is a major risk factor for developing cancer not only in colonic mucosa but also in other mucosal surfaces, namely, the oral cavity. Thereby, chronic inflammation emerging in colitis and periodontal disease may predispose individuals to colon and oral cavity cancer, respectively.

Consequently, several reports suggest a complex regulation by ATG proteins during carcinogenesis in colon and oral cavity. For instance, ATG16L1 mRNA levels were found to be supressed in both colon tumor samples as well as in healthy adjacent tissue [9]. In contrast, ATG16L1 mRNA was found to be strongly induced in response to a colibactin‐producing *E. coli* strain, which is associated with colon carcinogenesis [10]. Intriguingly, genetically‐prone mice to colorectal cancer lacking *atg16l1* in epitheial cells (*Apc* ^Min/+/^atg16l1^ΔIEC^) were more resistant to colon cancer if left uninfected, however, when these mice were infected with colibactin‐producing *E. coli* , those animals acquired a susceptible phenotype [10]. These data confirm that the role of ATG16L1 in colon cancer is influenced by several factors interacting with ATG16L1 mutations, as previously reported for colitis [11]. In terms of oral cancer, Wu et al. reported that samples from mice chemically‐induced with oral squamous cell carcinoma (OSCC) with 4‐nitroquinoline‐1‐oxide (4NQO), overexpressed markers for autophagy with a positive association between autophagy and advanced cancer stages [12]. Supporting this latter, Tang et al. reported abundant presence of ATG16L1 in tumor samples from human OSCC Likewise, ATG16L1 polymorphisms are associated with a variety of human cancers [13, 14, 15], but also with better prognosis in cancer [16]. Thus, the role of ATG16L1 in cancer seems to be more complex than previously thought and understanding a complete landscape of cellular processes modulated by ATG proteins is necessary.

ATG proteins act as important regulators during cancer development, and although their role in innate cells has been widely described, less is known about how immune adaptive cells are influenced by ATG proteins. Therefore, it remains to be addressed whether ATG proteins can modulate adaptive cells like B cells in the context of cancer, since this cell lineage represents an important checkpoint in the immune response by playing regulatory roles such as secreting abundant regulatory cytokines (e.g., IL‐10) and expressing membrane‐bound suppressive ligands such as PDL1 and FasL [17]. Previous literature showed that ATG proteins indeed modulate innate‐like B cells. Miller et al. reported an impaired ontogenic flux in peritoneal B1 cells in absence of ATG5, wherein ATG5 deficiency resulted in lower percentages of peritoneal B1 cells completing the transition from pro‐ to pre‐B cell [18]. Consequently, B cells might also be affected by other ATG proteins and thereby contribute to diseases like cancer; thus, we aimed to explore a role for ATG16L1 during carcinogenic processes by employing chemically induced preclinical models of solid tumors in colon and oral tissues and putative altered function displayed by B cells.

## 2 Material and Methods

### 2.1 Mice and Cancer Models

Eight‐to‐nine‐week‐old female wild type (WT) and ATG16L1 hypomorphic (ATG16L1^HM^) mice (mouse colony kindly donated by Dr. Herbert W. Virgin, University of St. Louis Washington), both in C57BL/6 genetic background, were used throughout the study. Animal colonies are bred and maintained at our faculty's mouse facilities and the use of these colonies was approved by the Bioethics committee and met the recommended guidelines issued by government for experimental animal care (NOM‐062‐ZOO‐1999).

The AOM/DSS model of colitis‐associated colorectal cancer (CAC) was implemented with slight modifications from the original protocol [19] given our colony of ATG16L1^HM^ mice is highly susceptible to intestinal inflammation (unpublished data). Mice were intraperitoneally (ip.) injected with the carcinogenic agent azoxymethane (AOM, Merck, Darmstadt, Germany) at 12.5 mg/kg (5 days prior to DSS delivery) and followed by three cycles of intestinal inflammation consisting of 5 days drinking DSS‐supplemented water (1.5%, MW 40 000 Thermo Scientific, USA) followed by 16 days of access to tap water without DSS. At the end of the third cycle, mice were additionally maintained drinking tap water for 8 weeks until sacrifice.

The widely used oral squamous cell carcinoma (OSCC) model was chemically induced by administering 4‐nitroquinoline‐1‐oxide (4NQO, Merck, Darmstadt, Germany) at 0.01% in drinking water for 16 weeks as previously reported [20].

### 2.2 Macroscopic Examination

In both experimental cancer protocols, bodyweight changes were recorded throughout the study. At sacrifice, corresponding tissues (i.e., colon and tongue) were excised in order to carry out a macroscopic visual assessment to observe apparent lesions and tumor presence as well as to measure tissue length. In CAC‐induced animals, colon samples were excised, washed in sterile PBS, and upon length assessment, tissues were longitudinally sectioned and photographed under a stereoscopic microscope. The tumor size and numbers were determined for each mouse and classified depending on their size as tumors no longer than 2 mm (< 2 mm) and tumors longer than 2 mm (> 2 mm). For OSCC‐induced animals, saliva samples were obtained at indicated times and tongue tissues were carefully removed and washed with sterile PBS. Macroscopic signs such as leukoplakia or tumors indicated the stage of carcinogenesis and are shown in representative images in Figure 4c.

### 2.3 Histopathology

Colon and tongue samples were collected as described and immediately fixed in 4% PBS‐buffered formalin (Fermont, México) for at least 24 h. Next, formalin was removed by thorough washes in running water for 30 min, and samples were dehydrated with increasing concentrations of alcohol. Tissues were embedded in paraffin (Paraplast, Leica Biosystems Pty. Ltd. Melbourne, Australia), and 5 μm‐thick sections were H&E (Hycel, México) stained and images captured under light microscope. Representative images from each group are shown. Quantitative analysis of the histological tongue sections was assessed as follows: 0; no dysplasia, 1; mild dysplasia, 2; moderate dysplasia, and 3; severe dysplasia.

### 2.4 Blood Samples

In order to determine circulating profile of total IgG levels, blood samples were collected by conducting tail snips at indicated times. The obtained blood samples were collected in sterile plastic tubes (Eppendorf Germany) containing 40 μL of anticoagulant (0.5 mM EDTA, J.T. Baker ThermoFisher Scientific, USA), which were subsequently centrifuged (3500 rpm/10 min) and plasma samples were collected and maintained at −80°C until used.

### 2.5 Cell Culture

Respective secondary lymphoid organs, mesenteric lymph nodes (MLN) and submandibular lymph nodes (SMLN), and spleen were removed at sacrifice and passed through a sterile 70 μm pore nylon mesh cell strainers (Falcon, Corning Inc. NY, USA). RBCs were depleted with ammonium chloride (J.T. Baker ThermoFisher Scientific, USA) ACK buffer and leukocyte suspensions seeded in complete RPMI 1640 medium (Cytiva, HyClone Laboratories, Utah, USA) at density of 3 × 10^6^/well in flat bottom 24‐well plates (Gibco, ThermoFisher Scientific, USA). Cells were stimulated with 5 μg of concanavalin A (Sigma‐Aldrich, USA) for 48 h and cell culture supernatants were collected and stored at −80°C until used for ELISA assays.

### 2.6 Elisa

The levels of IL‐4, IL‐15, and IFNγ in cell culture supernatants were determined following manufacturer's instructions (Peprotech, Rockhill NJ, USA). Total IgG levels were determined as follows: plasma samples from experimental groups were diluted in sterile albumin‐containing PBS (1:10) in flat bottom 96‐well plates (Costar Corning Inc. NY, USA). After overnight incubation at 4°C, plates were washed and blocked with 300 μL of bovine serum albumin (BSA, PeproTech proteins, Thermo Fisher Scientific, USA)‐containing PBS (1%) each well. A second round of washes was conducted, and plates were incubated (2 h, room temperature) in presence of HRP‐coupled anti‐mouse IgG (BioLegend Inc., CA, USA) diluted 1:5000 in BSA‐containing PBS (1%). After six washes, plates were developed by adding HRP substrate (3% hydrogen peroxide) and optical density (O.D.) was determined in a 96‐well plate reader (450 nm), and upon subtracting O.D. from blank wells, circulating total IgG levels present in plasma samples from experimental groups are shown.

### 2.7 Flow Cytometry

At indicated times, cells were recovered from control and cancer‐induced animals. Total cells from draining lymph nodes, MLN, and SMLN from mice induced with CAC and OSCC, respectively, as well as spleen from both models were obtained. Cell suspensions were generated by removing RBCs when required and, upon counting, cells were washed in flow cytometry buffer (1% FBS, 0.05% sodium azide in PBS) and adjusted to 1 × 10^6^/mL to proceed staining. Cell samples were incubated 30 min at 4°C with the combinations of fluorochrome‐conjugated antibodies for B cells (APC‐Cy7‐CD3 and PE‐CD19 and viability dye PerCP‐7AAD All from BioLegend Inc., CA, USA). The events were acquired in Attune NXT and analyzed with FlowJo V10 software (BD Biosciences OR, USA); representative plots gated on singlet and live cells (gating strategy in Figure 2b) are shown.

### 2.8 Bioinformatics Analysis

In order to seek any correlation between our findings and human studies, we conducted a search in the available NCBI Geo Datasets by using the keywords B cells, signature and cancer. We found a report by Chen et al., wherein peripheral blood B cells from healthy donors (*n* = 7) and breast cancer patients (*n* = 8) were sorted to perform a transcription profile [21]. Although ATG proteins were not the scope of this report (accession key GSE180285), the available raw data included several ATG proteins which could be compared. Thus, we grouped these data as healthy donors and breast cancer patients to compare ATG5, ATG12, ATG16L1, and ATG16L2 by using the GEO2R software to carry out a Limma (Linear Model for Microarray Analysis). The resulting analysis is shown in Figure S1.

### 2.9 Data Presentation

Data were analyzed by unpaired student's *t*‐test with Welch's correction, where *p* < 0.05 was accepted as significantly different. Data shown are mean ± SEM, and analysis was conducted by using the GraphPad Prism V9 software (Graph Pad Software MA, USA).

## 3 Results

### 3.1 ATG16L1 Hypomorphic Mice Are More Susceptible to CAC

**FIGURE 1:**
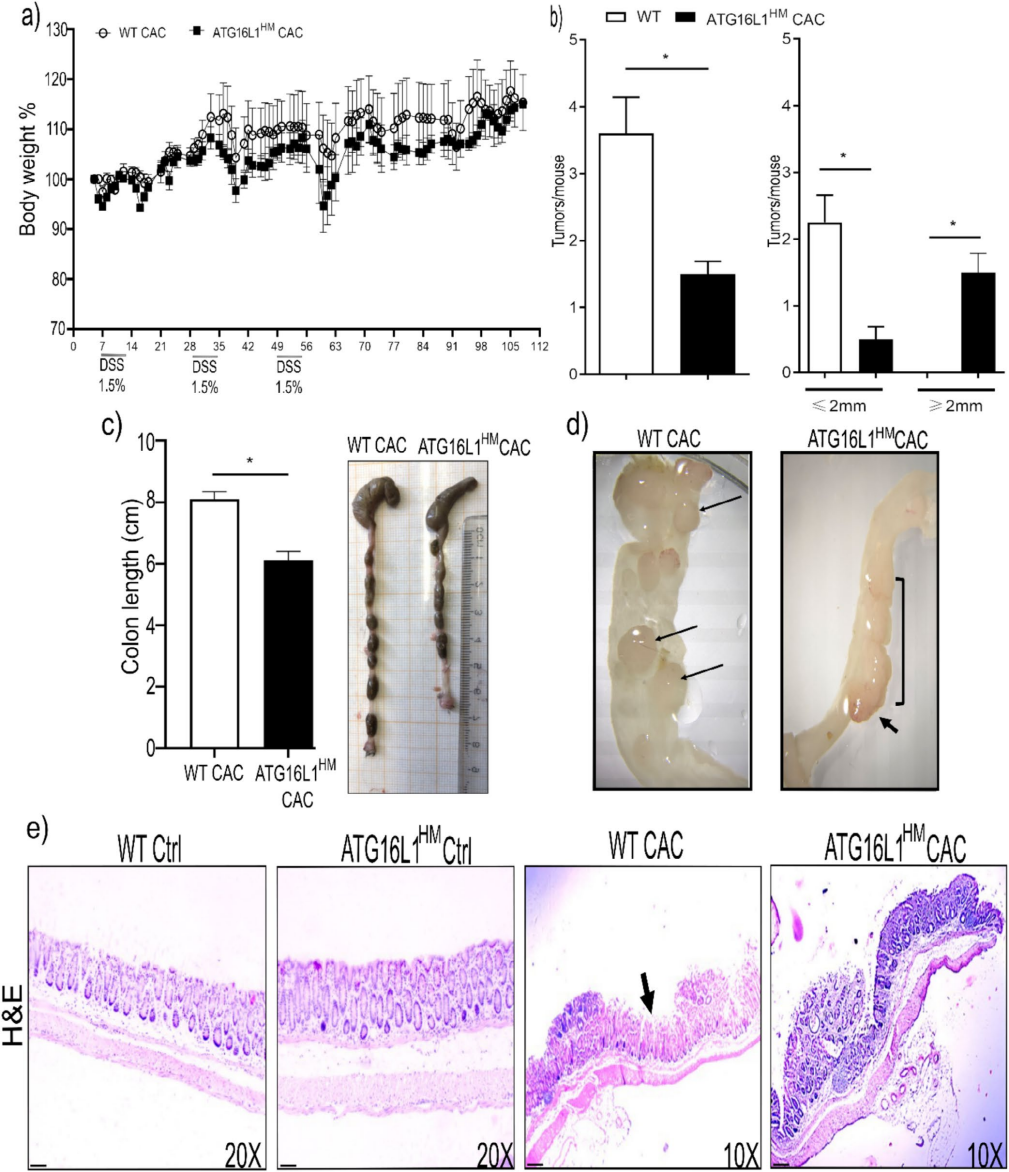
ATG16L1 protein contributes to cancer resistance in colonic mucosa. WT and ATG16L1^HM^ mice were induced with CAC and macroscopic and histopathological parameters were compared between groups. Bodyweight changes were recorded, and ATG16L1^HM^ mice exhibited a trend to lose more weight than WT littermates (panel a). Colon samples were collected at the end of the experiment and length was determined; ATG16L1^HM^ mice presented greater colon shortening and caecum damage as compared to their WT counterparts as shown in panel (b). In (c), colon tissues were longitudinally sectioned and evident tumors were observed, where WT animals presented individual tumors whereas in ATG16L1^HM^ mice mass of tumors were observed, evidencing an uncontrolled carcinogenic process in the ATG16L1^HM^ group. Panel (d) shows the number of tumors as well as tumor size, utilizing a 2 mm threshold for each tumor. Panel (e) shows representative H&E stained longitudinal sections from experimental groups, in which, WT group presented smaller tumors with incompletely transformed mucosa (arrow), whereas colon samples from ATG16L1^HM^ mice show continuous and larger transformed areas (The scale bar indicates 50 μm). Data shown are representative from three independent experiments (*n* = 9–10 each group) where mean ± SEM is depicted and * indicates *p* < 0.05 when student's *t*‐test was conducted.

To address the role of ATG16L1 in preclinical models of cancer, we utilized the AOM/DSS model of CAC and compared the parameters attributed to this disease between WT and ATG16L1^HM^ mice. We first observed fluctuations in bodyweight after DSS consumption, where ATG16L1^HM^ animals showed a non‐significant difference when compared to the WT group (Figure 1a). However, when colon tissues from experimental groups were excised, those from ATG16L1^HM^ mice presented a significant reduction in length relative to WT animals similarly induced with CAC (6.1 ± 0.7 versus 8.1 ± 0.6, respectively, Figure 1b). Also, rectal bleeding and prolapse were observed in approx. 50% of the ATG16L1^HM^ mice, whereas only 20% of WT animals exhibited these signs (data not shown). Subsequently, colon tissues were longitudinally sectioned and observed under a stereoscopic microscope and, as expected, WT mice presented individual small and large tumors (Figure 1c, left panel). In contrast, we observed that the vast majority of ATG16L1^HM^ mice (~90%) presented mass or fused tumors extended along the colon with highly vascularized areas (Figure 1c), suggesting a more severe course of CAC when ATG16L1 is not expressed as required. Moreover, histopathological analysis showed no altered architecture in colonic mucosa from both WT and ATG16L1^HM^ age‐ and sex‐controlled mice (Figure 1d, upper panels). Conversely, WT mice induced with CAC presented tumors in the colonic mucosa intercalated with zones of incomplete transformed tissue, whereas in histologic sections from ATG16L1^HM^ mice we observed larger tumors and continuous transformed colonic mucosa (Figure 1d, lower panels). Thus, mice with impaired ATG16L1 expression showed more widespread areas of transformed colonic epithelium, indicating that ATG16L1 limits tumor growth in CAC.

### 3.2 Susceptibility in ATG16L1 Hypomorphic Mice Is Accompanied With Differential Cytokine Release and Increased B Cells in Secondary Lymphoid Organs

**FIGURE 2:**
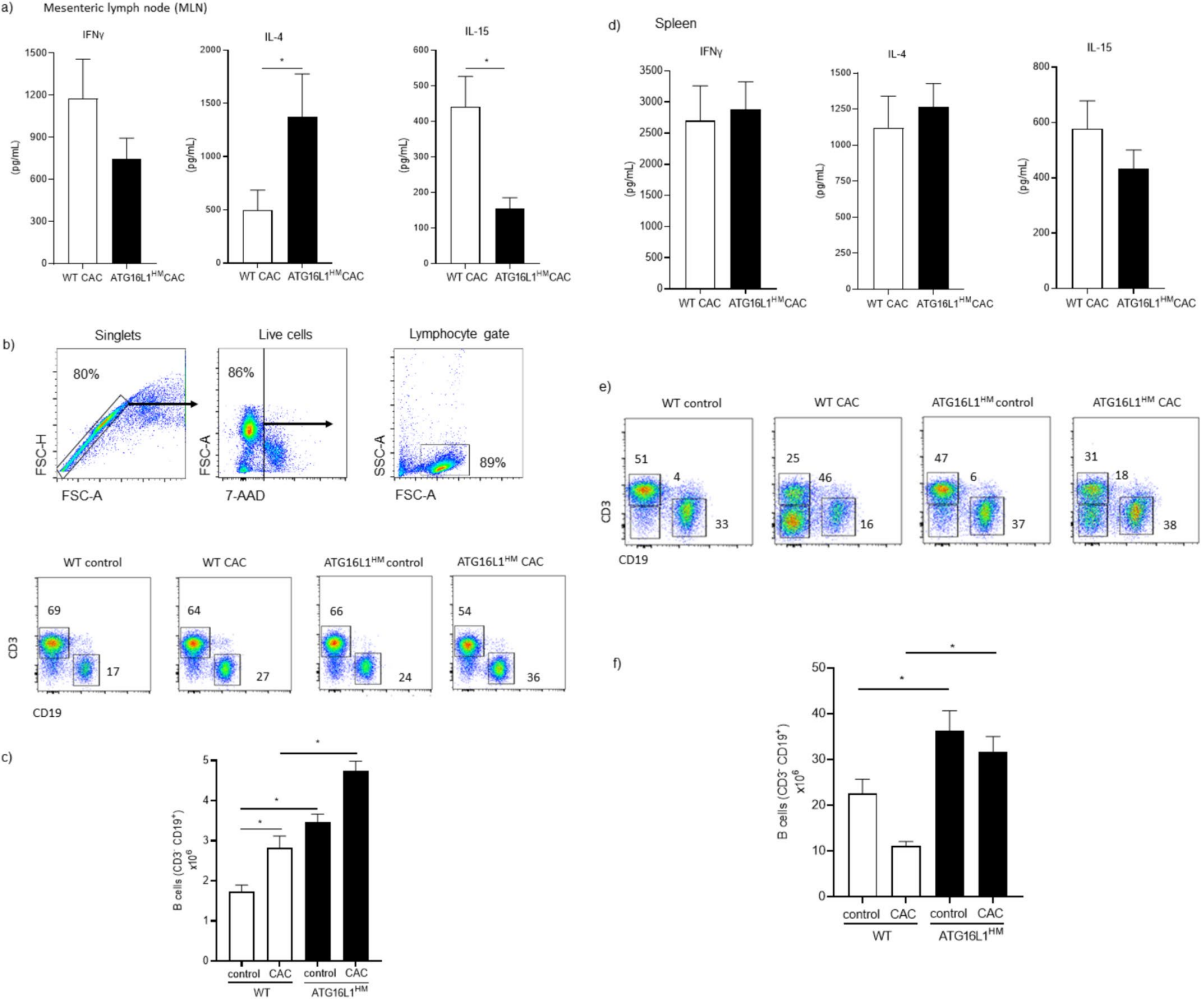
Susceptible ATG16L1^HM^ mice developed an altered immune response in secondary lymphoid organs upon colon cancer induction. MLN cells from WT and ATG16L1^HM^ mice were stimulated with the mitogen con A and cytokine output was determined in supernatants. Panel (a) shows cytokine levels in MLN culture supernatants wherein ATG16L1^HM^ mice predominantly produced Th2‐type cytokines (IL‐4) contrary to a reduced release of the immuno‐stimulating cytokine IL‐15. Additionally, cells were characterized by flow cytometry using the gating strategy shown in (b) and we found that ATG16L1^HM^ mice presented more B cells in MLN when compared to WT animals (panel b). When absolute numbers were determined based on total cell counts (panel c), ATG16L1^HM^ mice presented a significant increase in B cells both in control and under CAC‐induced conditions. Panel (d) shows cytokine release assayed in spleen cell culture supernatant, where no significant changes were noticed between experimental mice, and panels (e, f) show representative dot plots of splenocyte flow cytometry assay, in which ATG16L1^HM^ mice consistently had more B cells. Data shown are from three independent experiments (*n* = 6–8 each group), with similar results where mean ± SEM is depicted and * indicates *p* < 0.05 when student's *t*‐test was conducted.

During carcinogenesis, progressive changes in the immune response including a switch in cytokine production are observed. Here we quantified levels of IFNγ and IL‐4 as indicative of Th1/Th2 dichotomy and IL‐15 as an important cytokine involved in promoting innate (NKs) and adaptive (CD8^+^ T cells) cytotoxic cell survival [22, 23]. We found that cells collected from MLN in CAC‐induced WT mice produced higher, although non‐significant, levels of IFNγ compared to those levels found in supernatants from ATG16L1^HM^ mice (1178 ± 278 vs. 747 ± 146 pg/mL, respectively, *p* = 0.1) (Figure 2a). In contrast, cells from ATG16L1^HM^ mice produced significantly higher levels of IL‐4 as compared to their WT counterparts (1376 ± 400 and 496 ± 188 pg/mL, respectively, *p* = 0.04) (Figure 2a). Importantly, cells from ATG16L1^HM^ mice secreted reduced levels of IL‐15 in comparison to WT animals (155 ± 30 and 441 ± 84 pg/mL, respectively, *p* = 0.005) (Figure 2a). Hence, ATG16L1^HM^ mice, which are more susceptible to CAC, presented a local Th2‐biased immune response along with strongly reduced IL‐15 secretion, which partly explains why ATG16L1^HM^ mice are more permissive to colon tumor growth than WT organisms.

We also aimed to determine lymphocyte distribution in MLN from both WT and ATG16L1^HM^ mice induced with CAC. Cell suspensions were prepared, and the analysis of single and live cells followed by the gating strategy shown in Figure 2b. First, upon segregation from T lymphocytes (CD3^+^CD19^−^ cells), B lymphocytes were easily visible in both WT and ATG16L1^HM^ mice induced with CAC, which accumulated more B cells in MLN than mice from the control group in each strain (Figure 2c), but interestingly, we observed that ATG16L1^HM^ mice presented higher percentages of B cells in MLN related to WT mice similarly induced with CAC, and this difference was further sustained with the amount of absolute numbers of B cells determined in both groups (Figure 2c). Thus, restricted expression of ATG16L1 in mice results in over‐representation of B cells in MLN, under steady conditions and upon CAC induction.

We additionally sought the previously mentioned cytokines (IFNγ, IL‐4, and IL‐15) and B cell numbers in splenocytes harvested from both experimental groups. In this case, we did not observe significant differences between WT and ATG16L1^HM^ groups in terms of the cytokines assayed when quantified in splenocyte culture supernatants, suggesting that cytokine profiles were selectively modified in local draining lymph nodes (Figure 2d). In regard to B cell numbers in the spleen, in contrast to findings observed in MLN where CAC induction caused enlarged B cell populations as compared to control animals, we found that WT mice with CAC presented reduced B cell populations (Figure 2e,f). Interestingly, in susceptible ATG16L1^HM^ mice the numbers of B cells were not reduced in mice induced with CAC when compared to control groups. Also, a significant difference was observed between the B cell population in the spleen from CAC‐induced WT mice related to B cells present in ATG16L1^HM^ mice with CAC (Figure 2f). Strikingly, our flow cytometry analysis revealed that in the spleen, unlike MLN, a third population of lymphocytes double negative (CD3^−^ CD19^−^) accumulated; however, ATG16L1 mutant mice had reduced percentages of such population compared to those found in the WT group. We currently are exploring this population in order to determine their identity as well as their likely contribution to the CAC progression; a putative NKTs or NKs phenotype is suspected.

**FIGURE 3:**
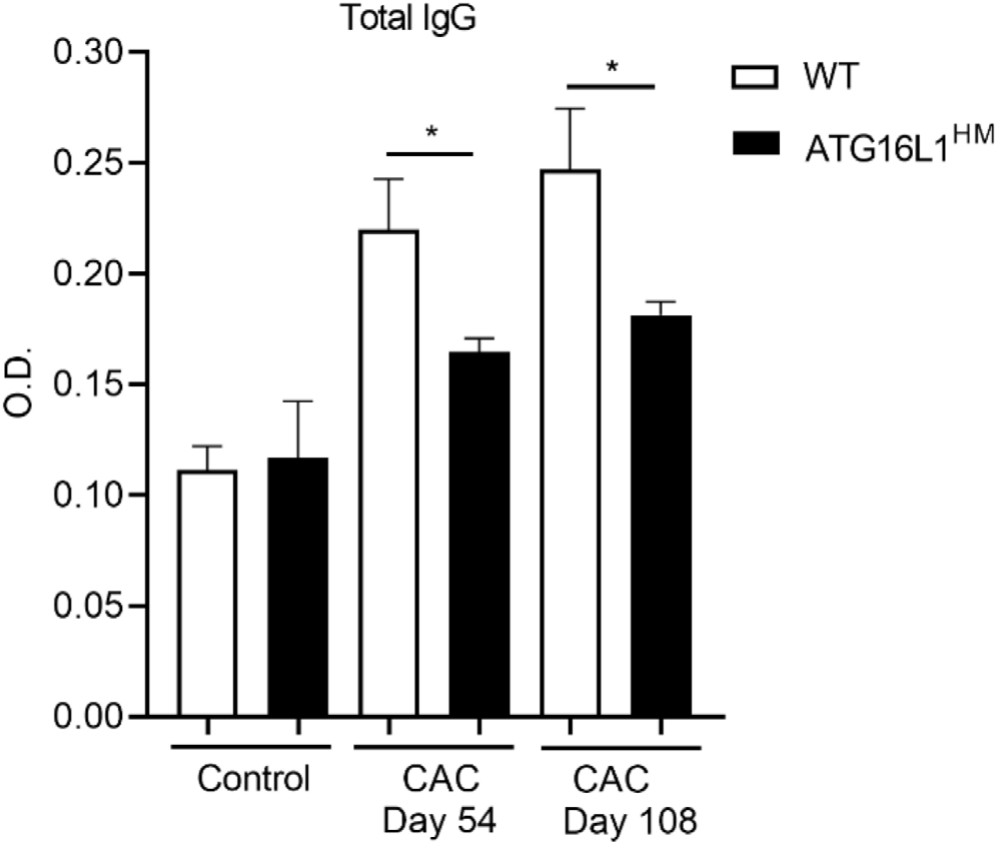
Hypomorphic ATG16L1 mice secreted reduced levels of antibodies. At indicated times, blood samples were collected from both groups to determine total IgG levels, and optical density is shown. Data are from three experiments (*n* = 7–8 each group), and mean ± SEM is depicted where * indicates *p* < 0.05 using student's *t*‐test.

The striking difference in B cell numbers suggested further defects in these cells; therefore, we quantified circulating total IgG levels in WT and ATG16L1^HM^ mice induced with CAC. ELISA assays revealed that the induction of CAC in WT mice resulted in time‐dependent increased plasma levels of IgG, whereas in mutant ATG16L1^HM^ mice the IgG production was significantly affected (Figure 3). Therefore, highly susceptible ATG16L1^HM^ mice harbored more B cells in secondary lymphoid organs, but these cells may present impaired antibody secretion.

### 3.3 The Anti‐Oncogenic Features of ATG16L1 Are Extended to Oral Mucosa

**FIGURE 4:**
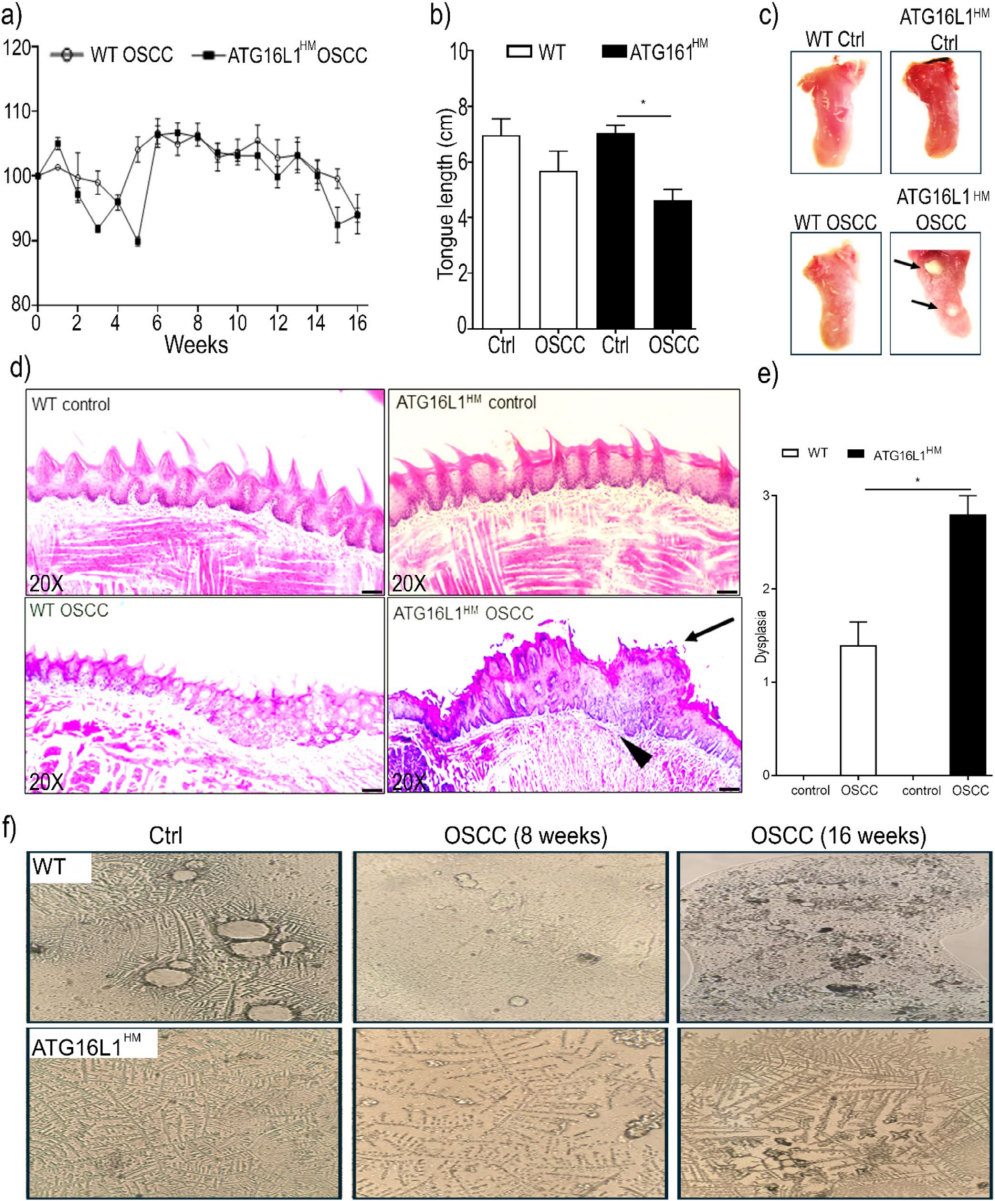
ATG16L1 hypomorphic mice are more susceptible to neoplasia in oral mucosa. We induced experimental OSCC and compared distinct parameters between WT and ATG16L1^HM^ mice. Bodyweight changes were weekly monitored during administration of 4NQO and are reported in (a). Tongue length was assessed with a digital vernier and is shown in panel (b) with representative images of these tissues also shown in (c), where arrows point to tumors found in ATG16L1^HM^ mice, whereas leukoplakia was observed in WT mice. Representative histopathological H&E stained sections are shown in (d), where samples from strain‐matched control mice show intact fungiform papillae with unaltered architecture (upper panels). In contrast, in samples from animals exposed to 4NQO (lower panels), papillae are virtually absent in WT mice whereas in ATG16L1^HM^ severe dysplasia (arrow) and a likely impaired integrity in basal membrane (arrowhead, the scale bar indicates 50 μm). As described in methods, the extent of dysplasia was assessed and is shown in (e). In panel (f), saliva samples under light microscope with differences in mucin patterns are shown (*n* = 6 in control groups and *n* = 10 in 4NQO‐given groups) data are representative from three experiments and * indicates *p* < 0.05.

We decided to further explore whether the genetic impairment in ATG16L1 expression may be modulating carcinogenesis in other tissues and employed the OSCC mouse model. When WT and ATG16L1^HM^ mice were induced with oral cancer by means of exposure to 4NQO in drinking water as described in methods, we observed significant changes in bodyweight between groups only at certain time points such as weeks 3, 5, and 15, where ATG16L1 mutant mice presented diminished bodyweight records (Figure 4a). Additionally, we conducted a macroscopic assessment of tissues recovered from experimental animals including tongue length and lesions. Macroscopically, neither apparent lesions nor significant changes in tongue length were observed in samples from WT and ATG16L1^HM^ control groups (6.9 ± 0.5 vs. 7 ± 0.3 cm, respectively). In fact, for WT animals there was no significant difference between control mice and their counterparts induced with OSCC (Figure 4b). However, length measurements revealed a significant shortening of tongue collected from OSCC‐induced ATG16L1^HM^ mice as compared to tongue excised from ATG16L1^HM^ control mice (4.6 ± 0.5 vs. 7 ± 0.3, respectively), although the length recorded in OSCC‐induced ATG16L1^HM^ mice was not statistically different from their WT counterparts (Figure 4b,c). Furthermore, WT mice induced with experimental OSCC showed tissue thickening due to hyper keratinization processes (leukoplakia), whereas individuals from the ATG16L1^HM^ group exhibited tumors with variable sizes as shown in representative images (Figure 4c). Additionally, histopathological analysis carried out on H&E‐stained slides showed no signs of disease in both WT and ATG16L1^HM^ drinking tap water; however, in samples from WT animals induced with OSCC we observed dysplasia and hyper keratinization where a few papillae are still preserved and the basal membrane remained intact (Figure 4d). In contrast, an atrophic epithelium with hyperkeratosis is observed in ATG16L1^HM^ individuals (Figure 4d). Also, there seem to be zones with perforated basal membrane, suggesting that malignant cells might be infiltrating (Figure 4d). Thus, histological sections confirmed more advanced stages of OSCC in ATG16L1^HM^ when compared to WT animals. Intriguingly, saliva samples showed that, despite the toxic effect of drinking 4NQO, ATG16L1^HM^ mice presented a more preserved mucin‐forming pattern as relative to WT mice (Figure 4e). Our findings demonstrated that ATG16L1 exerts a tumor suppressor role in the oral mucosa similarly to what we observed in colon.

**FIGURE 5:**
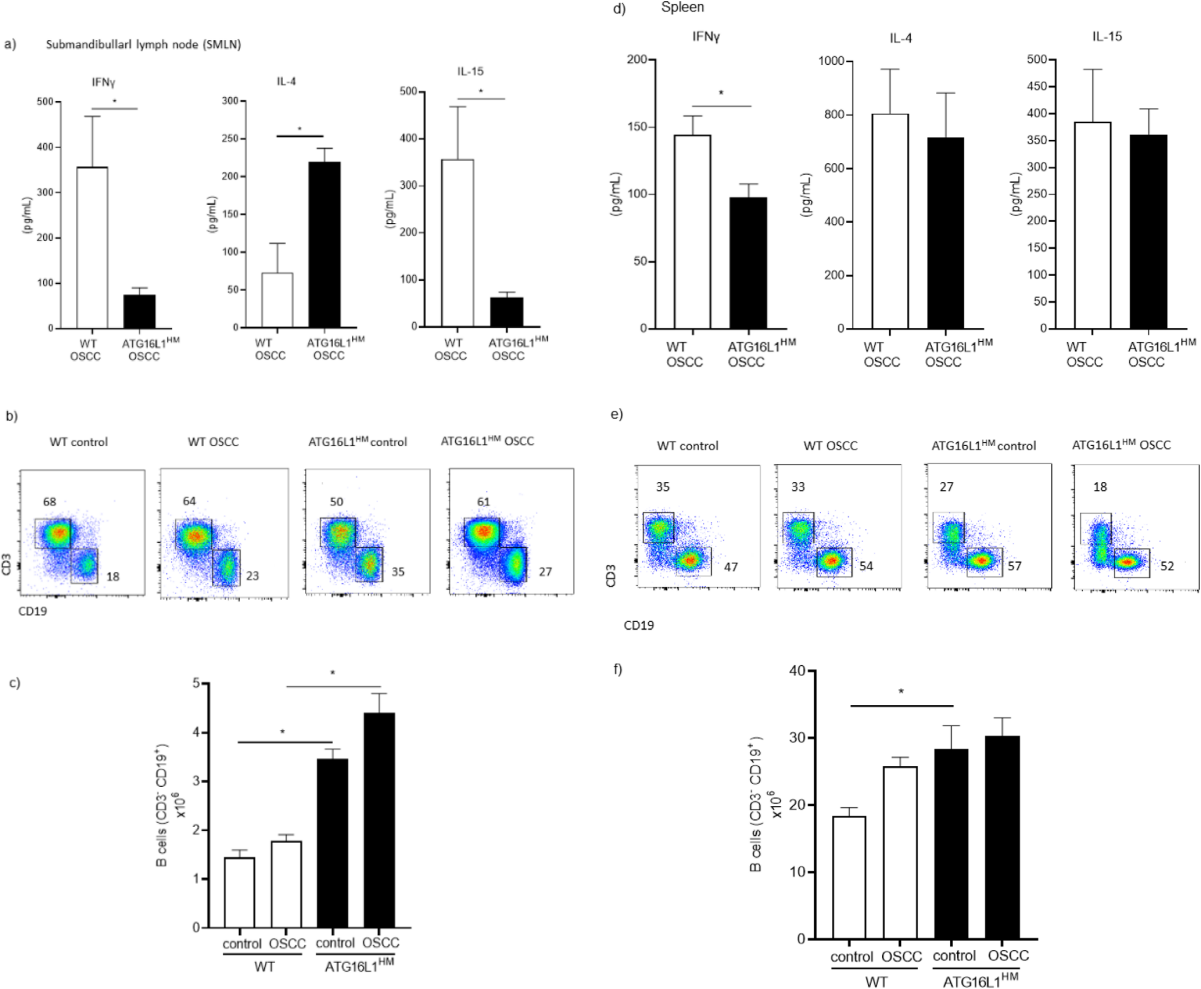
Oral cancer caused a Th2 response and increased B cells in permissive ATG16L1 mutant mice. Total cells from SMLN and spleen from WT and ATG16L1^HM^ mice were stimulated with the mitogen con A and cytokine output was determined in culture supernatants. Panel (a) shows cytokine levels in SMLN where IL‐4 dominated the local response, as observed in the CAC model. Cells also were characterized by flow cytometry and absolute numbers are shown in (b, c). Panels (d–f) show cytokine release and B cell populations assayed in local SMLN, where ATG16L1^HM^ mice presented a distinct cytokine profile and higher numbers of B cells as compared to WT animals. Data shown are from two independent experiments (*n* = 8), with similar results where mean ± SEM is depicted and * indicates *p* < 0.05 when student's *t*‐test was conducted.

**FIGURE 6:**
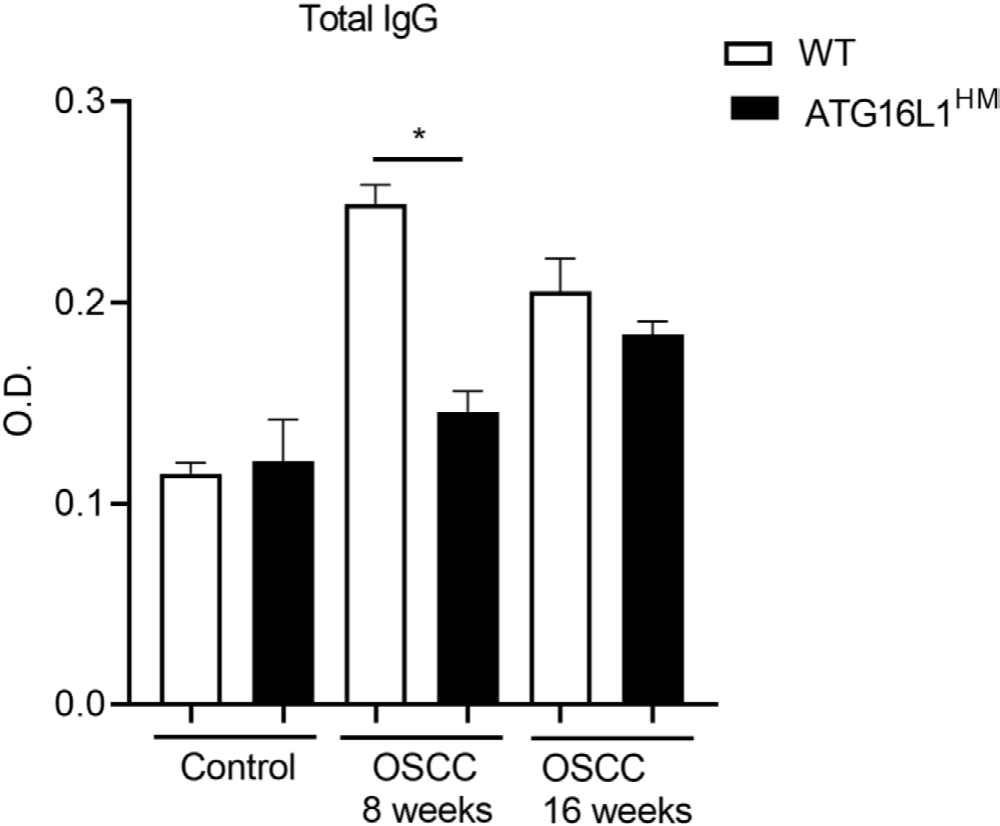
A reduced secretion of IgG antibodies is a common finding in hypomorphic ATG16L1 mice. Both WT and ATG16L1^HM^ mice were induced with OSCC as described in methods, and at indicated times blood samples were collected and assayed for total IgG antibodies as indicated in methods. Data are from two experiments (*n* = 4–6 each group), and mean ± SEM is depicted where * indicates *p* < 0.05 using student's *t*‐test.

We also determined cytokine levels in secondary lymphoid organs. Consistent with the findings in the CAC model, we observed a clear Th2 polarization in SMLN from ATG16L1^HM^ mice at the end of OSCC induction; this was evidenced by low levels of IFNγ and substantial release of IL‐4 compared to the levels of the same cytokines quantified in supernatants from SMLN cultures generated from WT animals with OSCC (Figure 5a). Likewise, SMLN cells from ATG16L1^HM^ mice showed a strongly impaired ability to secrete the immuno‐stimulant cytokine IL‐15, since SMLN cells from WT animals produced nearly a six‐fold increase in this cytokine upon incubation with the mitogen con A (63 ± 11 vs. 358 ± 103 pg/mL, respectively). Furthermore, when the B cell percent and absolute numbers were determined in the SMLN from experimental groups, we found a threefold increase in B cell numbers from SMLN cells collected from ATG16L1^HM^ mice compared to those found in WT animals (Figure 5b,c). Conversely, when cytokine levels were assayed in splenocyte culture supernatants, significant changes were only observed in IFNγ release, wherein spleen cells from WT animals produced more IFNγ, whereas IL‐4 and IL‐15 were comparable between WT and ATG16L1^HM^ mice (Figure 5d). In regard to splenic B cell populations, a significant difference was found only under steady conditions where ATG16L1^HM^ mice presented a greater percent of B cells, and these differences were sustained when absolute numbers were calculated, as compared to WT control mice (Figure 5e,f). Finally, we observed that IgG levels peaked at 8 weeks of OSCC induction; however, IgG levels in ATG16L1^HM^ mice were also found to be significantly reduced at this time (Figure 6). Although no significant changes in IgG output at the end of the OSCC model were found, this is attributed to a decrease in IgG release observed in WT animals rather than increased IgG production in ATG16L1^HM^ mice (Figure 6).

Therefore, as found in the CAC model, ATG16L1^HM^ mice turned out to be highly prone to develop oral cancer than their WT littermates and the mechanisms underlying this seem to be conserved, and these include a strong Th2 polarization and a defective B cell response.

## 4 Discussion

ATG proteins are involved in fundamental processes including both autophagy‐dependent and autophagy‐independent, and it is currently recognized that autophagy displays dual roles in cancer: by removing cellular stressors in early stages which reduces exposure of cells to insults that ultimately may trigger cancer, but autophagy also is an adaptive mechanism for tumor cells to survive harsh conditions such as hypoxia and other metabolic changes [24]. Indeed, previous studies described abundant presence of ATG16L1 in tissue samples from human patients with colon and oral cavity cancer; however, whether this protein exerted any role was not explored. Here, we identified that mice unable to express normal levels of the autophagic protein ATG16L1 presented a rapid progression of cancer, relative to mice with intact ability of expressing this same protein, concomitantly with an altered distribution of B cells in secondary lymphoid organs and lower levels of circulating IgG.

The role of ATG16L1 seems to be highly complex since recent studies reported that ATG16L1 promoted tumor growth in colon and liver metastasis [25] but also loss‐of‐function mutations in ATG16L1 were associated with improved prognosis in colon cancer [16]. Likewise, several reports describe that samples collected from oral and colon cancer present high expression of autophagy markers, including ATG16L1; however, it has been shown that the absence of other ATG proteins indeed results in spontaneous liver cancer [26]. Our results showed that hypomorphic ATG16L1 mice developed larger tumors than those found in WT animals in both colonic mucosa (Figure 1c,d) and tongue (Figure 4c,d). These data suggest that long‐term global deficiency in ATG16L1 is supporting uncontrolled cell transformation.

The accelerated carcinogenesis in ATG16L1^HM^ mice was accompanied by increased levels of the Th2‐related cytokine IL‐4, whose features include programming macrophages into a M2 profile which supports tissue remodeling and tumor growth [27]. This latter was not explored in this study, but it is highly possible to occur since previous reports stated that ATG proteins contribute to M2 polarization [28]. Furthermore, additional roles for excessive IL‐4 may also include the observed expansion of B cells, since IL‐4 promotes B cell proliferation [29]. The cytokine output assays also revealed that accelerated tumor growth in ATG16L1^HM^ mice was negatively associated with decreased production of IL‐15, a cytotoxicity‐promoting factor [23]. Thus, cytokine imbalance where increased Th2 polarization and impaired ability to secrete IL‐15, at least locally, shows that ATG16L1 might be targeting T cell differentiation. Whether this latter is a direct effect or through APC modulation remains to be explored. Moreover, the altered cytokine polarization between groups must be defined, since both IL‐4 and IL‐15 belong to the gamma‐common chain cytokine family, and whether they are produced by the same T cell population or distinct populations will help to further extend the knowledge on the several mechanisms regulated by ATG16L1 in cancer.

Also, ATG16L1^HM^ mice presented larger populations of B cells in secondary lymphoid organs than B cell populations found in WT animals. Increased B cells prior to cancer induction indicates that impaired expression of ATG16L1 results in either increased proliferation, increased infiltration, or defects in lymphatic organ egress. We are investigating this phenomenon since we also found more B cells in the peritoneal cavity as well (unpublished data), but unlike B cells from secondary lymphoid organs, B cells in the peritoneum expressed PDL1. Further, it has been shown that B cells undergo cellular metabolic adaptations during transition into plasma cells, including activation of autophagy [30]. Interestingly, it seems that ATG proteins display selective roles as evidenced by excessive antibody secretion [31] and impaired B cell ontogenic processes in absence of ATG5 [18]. This latter, however, contrasts with this study where we show that ATG16L1 limits B cell expansion (Figure 2) and is required for antibody release (Figure 3). In line with this, the analysis conducted on the datasets created by Chen et al. showed that sorted cells from breast cancer patients significantly increased the mRNA transcripts of ATG16L1 and ATG16L1, whereas ATG5 and ATG12 showed lower expression when compared to healthy donors ([21] and Figure S1). Strikingly, both ATG5 and ATG16L1 belong to the same ligase‐like complex collaborating for autophagosome elongation [32], but they seem to display opposite roles in B cell immunobiology. Determining this putative role of ATG16L1 on B cells will extend our knowledge on how ATG proteins regulate immune adaptive cells which are also important for vaccines.

Thus, in this report we show that ATG16L1 restrains tumor growth in distinct mucosal surfaces, which might be resulting from several defects including dysregulated cytokine polarization and B cell functions. Although our data show a central anti‐oncogenic role exerted by ATG16L1 some limitations are evident. For example, whether B cells with impaired ATG16L1expression are directly involved in tumor growth it remains to be determined, our data only show an association, reconstitution experiments with ATG16L1‐sufficient B cells should aid to answer this question. Also, there is a great variety of B cells with immune‐suppressive features, it is therefore urgent to uncover the phenotype of the B cells observed in this study, as well as heterogeneity within this population, in order to determine whether they are compatible with a previously reported B regulatory program or not. In an attempt to establish the translational potential of our study, it is necessary to identify whether these B cells express molecules against which approved immune‐therapies are designed. Furthermore, due to its imperative role in the autophagy process, it must also be determined whether the observed alterations in B cell populations are related to this process, the availability of autophagy inducers (rapamycin) as well as early (3‐methyladenine 3‐MA) and late (bafilomycin A1 and hydroxychloroquine) inhibitors of autophagy will contribute to explore this hypothesis in purified B cells. These questions are currently being addressed in our laboratory on purified B cells from distinct anatomic compartments.

## 5 Conclusion

Long‐term impaired expression of ATG16L1 causes more severe carcinogenesis in colonic and oral mucosa, resulting from a defective B cell response and a strong polarization toward a Th2‐type profile. Studies aimed to identify differential expression of autophagic proteins and its consequences in distinct cancer stages in humans are scarce. Understanding how ATG proteins regulate B cells during cancer development in humans will add in unveiling the potential therapeutic applications.

## Author Contributions

Investigation: Daniela Mendiola, Betsaida Ortiz, Oscar Nieto, Marisol I. González, Alejandro Martínez, Diego Mateos, and Mónica G. Mendoza‐Rodríguez. Formal analysis: Danielle Vannan, Bertus Eksteen, Miriam Rodríguez‐Sosa, Luis I. Terrazas, and José L. Reyes. Funding acquisition: José L. Reyes. Project administration: Marisol I. González. Writing – review and editing: Danielle Vannan, Bertus Eksteen, and José L. Reyes.

## Funding

This work was supported by the Dirección General de Asuntos del Personal Académico, Universidad Nacional Autónoma de México (DGAPA‐UNAM PAPIIT IN224520).

## Ethics Statement

Animal experiments met the recommended guidelines issued by government for experimental animal care (NOM‐062‐ZOO‐1999).

## Consent

The authors have nothing to report.

## Conflicts of Interest

The authors declare no conflicts of interest.

## Supporting information


**Figure S1:** Selective transcriptional regulation of ATG proteins in B cells from healthy donors and breast cancer patients. We conducted a comparison of the transcripts of various ATG proteins in B cells sorted from healthy donors (*n* = 7) and breast cancer patients (*n* = 8) from the study reported by Chen et al. as described in methods.

## Data Availability

The data that support the findings of this study are available from the corresponding author upon reasonable request.
