# Supplementary material for: Insufficient Expression of the Autophagic Protein ATG16L1 Results in Accelerated Carcinogenesis Related to an Aberrant B Cell Response
Source: Cancer Rep (Hoboken). 2026 Feb 9;9(2):e70438. doi: 10.1002/cnr2.70438 (PMC12885121; doi:10.1002/cnr2.70438)
Supplement: Supplementary file 1 — Figure S1: Selective transcriptional regulation of ATG proteins in B cells from healthy donors and breast cancer patients. We conducted a comparison of the transcripts of various ATG proteins in B cells sorted from healthy donors (n = 7) and breast cancer patients (n = 8) from the study reported by Chen et al. as described in methods. [file CNR2-9-e70438-s001.docx]

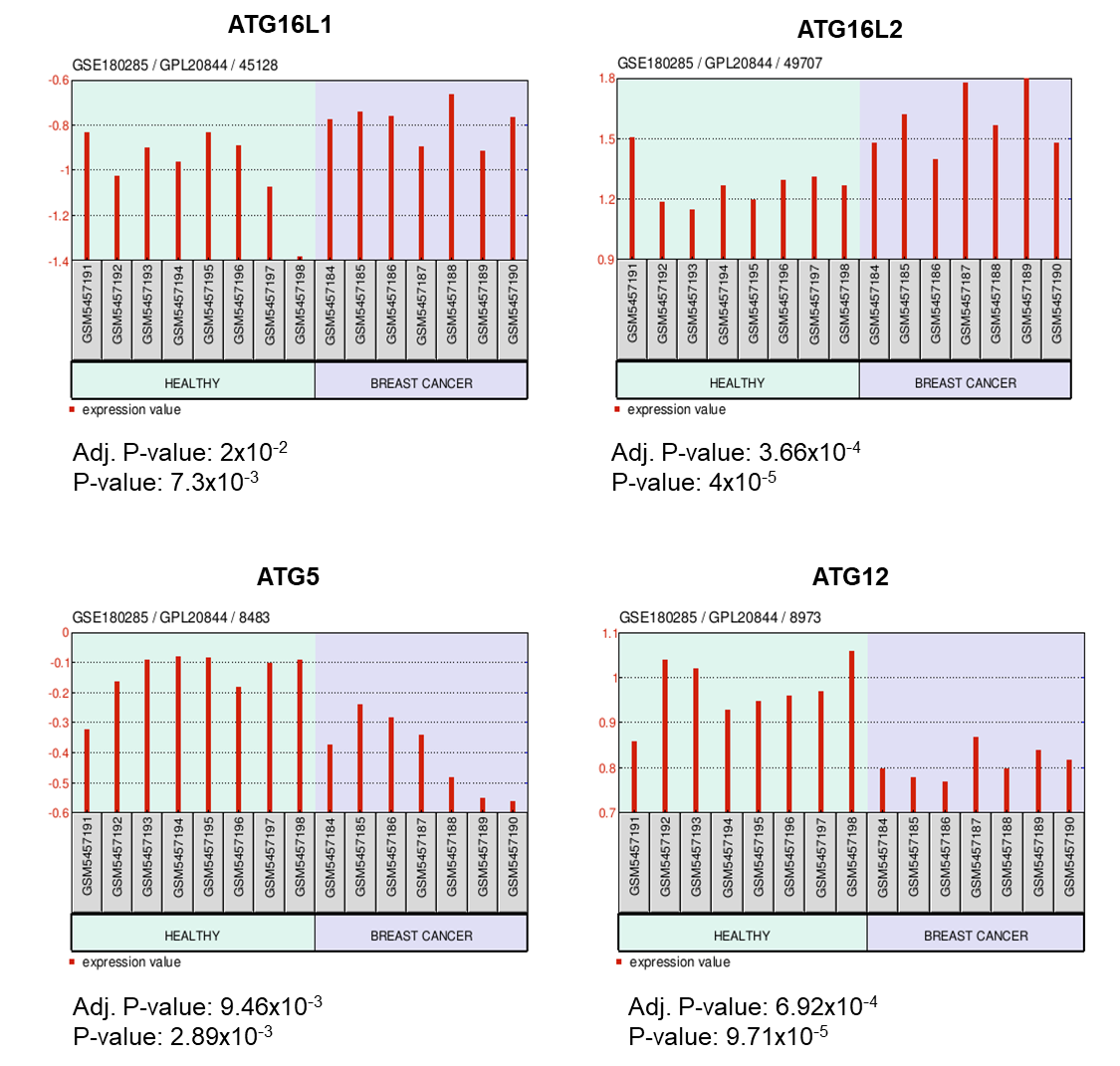


**Supplementary Figure 1.- Selective transcriptional regulation of ATG proteins in B cells from healthy donors and breast cancer patients.** We conducted a comparison of the transcripts of various ATG proteins in B cells sorted from healthy donors (n=7) and breast cancer patients (n=8) from the study reported by Chen *et al.* as described in methods.
